# The effect of four loading intravitreal aflibercept injections on macular fluid in treatment-naïve neovascular age-related macular degeneration

**DOI:** 10.1038/s41433-024-03214-x

**Published:** 2024-07-06

**Authors:** Shruti Chandra, Raffaele Raimondi, Alicia Lim, Amy Mohan, Sneha Melmane, Geeta Menon, Manju Chandran, Sobha Sivaprasad, Benjamin J. L. Burton, Ajay Kotagiri

**Affiliations:** 1 National Institute of Health Research Moorfields Biomedical Research Centre, Moorfields Eye Hospital, London, EC1V 2PD UK; 2 Institute of Ophthalmology, University College, London, EC1V 9EL UK; 3 South Tyneside and Sunderland NHS Foundation Trust, Sunderland, UK; 4 Frimley Health NHS Foundation Trust, Surrey, UK; 5 James Paget University Hospitals NHS Foundation Trust, Norfolk, UK

**Keywords:** Outcomes research, Business and industry

## Abstract

**Purpose:**

To evaluate the effect of four versus three loading aflibercept injections on macular fluid resolution and visual acuity (VA) in exudative neovascular AMD (nAMD).

**Methods:**

Multicentre, retrospective cohort study of treatment naïve nAMD eyes undergoing 3 versus 4 loading doses of aflibercept. Change in VA and fluid resolution on optical coherence tomography (OCT), were evaluated at 8 weeks post loading. The primary outcome was proportion of patients with no intraretinal (IRF) and/or subretinal (SRF) fluid at central 1 mm and whole macula at 8 weeks after loading. Data were summarised with mean ± SD for continuous variables, and *n* (%) for categorical variables.

**Results:**

Data from 995 patients was analysed (355 patients − 4 loading doses and 640–3 loading doses). At 8 weeks post 4 loading doses proportion of eyes with neither IRF nor SRF, no IRF and no SRF were 62.8%, 88.7% and 79.2% at fovea versus 56.1%, 87.9% and 69.9% in the whole macula, respectively. Fluid resolution at both fovea and macula were significantly higher in eyes with 4 loading injections versus 3 (*p* = 0.0001). The mean VA change was +4.0 (±11.3) and +5.4(±13.3) letters for 3 and 4 loading doses groups (*p* = 0.09).

**Conclusion:**

Four loading dose injections of aflibercept results in higher proportion of eyes with total fluid resolution in the central subfield and total macular scan when compared to those receiving 3 loading dose injections at 8 weeks post loading phase. However, the better drying effect of 4th loading dose does not translate into better short-term VA outcomes.

## Introduction

Aflibercept is widely used to treat exudative neovascular age related macular degeneration (nAMD) [1]. Based on the VIEW studies, aflibercept therapy is initiated as a loading phase of monthly intravitreal doses for 3 months. The treatment interval is then extended to 8-weekly dosing. This regimen is substantiated by data that show that the mean (±SD) duration of aqueous VEGF suppression with aflibercept is 67 ± 14 days [2]. In the VIEW studies at week 12 (4 weeks after 3 loading doses), macular fluid on time-domain OCT was present in 44.5% of patients on ranibizumab, 29.8% and 33.5% in the 2q4 and 2q8 of the aflibercept arms respectively [3]. However, the visual outcome at this timepoint did not differ between arms or between eyes with and without SRF or IRF.

In the real-world, macular fluid status across the entire 6x6mm macular scan rather than fluid in the central sub-field is usually considered to determine re-treatment strategies. The proportion of patients with macular fluid at week 12 (4 weeks after 3 loading aflibercept injections) in real-world is lacking due to the aflibercept label of reviewing patients 8 weeks following 3 loading doses [4–6]. This time-point is now important due to the availability of new anti-VEGF agents and ranibizumab biosimilars. Ranibizumab biosimilars are initiated as monthly injections and the consensus is to switch to an agent with better macula drying effect if there are signs of disease activity 4 weeks after 3 loading doses [7, 8].

In addition, the effect of a fourth loading aflibercept injection is not known.

This has become important now because of the trial design of TENAYA and LUCERNE that evaluated the role of faricimab in exudative nAMD [9]. Aflibercept was used as a comparator, but patients randomised to the faricimab arm received four loading injections compared to the patients in the aflibercept arm that received three initial injections and the aflibercept arm moved on to a fixed dosing interval of 8 weeks [9]. It is unknown whether a fourth aflibercept injection added to the loading phase would have made a difference to the outcomes at 20 weeks (8 weeks post 4 loading injections) [9]. A like for like comparison of 4 loading doses of aflibercept versus faricimab could not be done due to the study design.

In clinical practice during the COVID-19 pandemic, some clinicians decided to review the patients at the earliest opportunity although the majority were still reviewed around 2 months post last loading injection [10]. This provided an opportunity to compare the macular fluid outcomes at 8 weeks following 4 aflibercept injections versus 8 weeks after 3 aflibercept injections.

In this study, we aimed to evaluate the effect of the loading phase of aflibercept injections on macular fluid resolution in exudative nAMD in three ways. Firstly, we compared macula fluid status in eyes that were reviewed at 4 weeks after 3 aflibercept loading doses (week 12) and compared to the VIEW studies while the outcomes of fluid status in central subfield at week 12 were compared to the aflibercept arm in TENAYA and LUCERNE trials. Secondly, we evaluated the effect on fluid resolution at 8 weeks after 4 versus 3 monthly loading doses of aflibercept. Thirdly, we compared the visual outcome at 8 weeks after 4 versus 3 monthly loading doses of aflibercept.

## Methods

### Study design

Data on this retrospective cohort was extracted from the Electronic Medical records and Spectralis OCT (Heidelberg Engineering GmbH, Heidelberg, Germany) from 4 centres across the United Kingdom (UK). This service evaluation was approved by the clinical effectiveness department (CA23/MR/1234). Data was collected from December 2019 to August 2021. Inclusion criteria were consecutive patients with treatment naïve nAMD who underwent 4 loading aflibercept injections from 2 sites and with VA and OCT done at 12 and 20 weeks after first aflibercept injection. A cohort from 2 more sites that received 3 loading injections and reviewed 8 weeks post loading (i.e., at 16 weeks after 1st aflibercept injection) was included as a comparator.

Exclusion criteria were co-existent ocular disease that, in the opinion of the investigator, could affect or alter VA during the study, poor image quality and missing baseline OCT scans.

### Data collection

In addition to age, gender, ethnicity (White, Black, South Asian, other Asian and other), visual acuity records and frequency of loading injections (4 versus 3 injections) were obtained from electronic medical records. The Heidelberg Spectralis OCT parameters included central subfield thickness (CST) and the macula raster scans (6x6mm) were graded for presence or absence of IRF, SRF in the whole macula scan and central subfield. The CST was defined as average retinal thickness in the central 1 mm of ETDRS grid of a fovea centred scan. It was measured from inner boundary of internal limiting membrane to the outer boundary of Bruchs membrane and therefore included fovea-involving pigment epithelial detachments [11]. The SRF was defined as presence of hyporeflective space between the neurosensory retina (NSR) and the underlying retinal pigment epithelium (RPE) on OCT [12] while IRF was defined as presence of increased retinal thickening associated with a reduction in retinal tissue reflectivity on OCT [13]. Absence of SRF or IRF was assessed across the whole macular scan (6 × 6 mm) and at subfoveal location (1 mm central subfield).

### Outcomes

Outcomes included the proportions of patients in 4 loading injections group with no IRF, no SRF and neither IRF nor SRF (12 weeks and 20 weeks post-first aflibercept injections. These results were compared to the outcomes at 4 weeks post 3 aflibercept loading injections versus TENAYA and LUCERNE trials (central 1 mm subfield). In addition, the OCT outcomes at 8 weeks after 4 loading aflibercept injections versus 3 loading aflibercept injections were compared. The VA outcomes were also compared between the two loading regimes −3 injections versus 4 injections: 8 weeks post LP completion i.e., 16 and 20 weeks respectively.

### Statistical analysis

Data were summarised with mean ± SD for continuous variables, and *n* (%) for categorical variables. Eyes were divided into 2 groups for comparison analysis based on number of loading doses received. Chi-square test was used to explore differences in proportions among categorical data in independent groups. *P-*values < 0.05 were considered statistically significant. Data was analysed using SPSS Statistics for Windows, Version 24.0. Armonk, NY: IBM Corp.

## Results

### Study population and baseline characteristics

Of the 1104 patients included in the study, 995 eyes of 995 patients were analysed. The study cohort consisted of 355 eyes of 355 patients who received 4 loading doses of aflibercept and 640 eyes of 640 patients who received 3 loading doses of aflibercept. The flow of participants in shown in Supplementary Fig. S1. The demographic and baseline clinical characteristics for both groups are shown in Table 1. The mean age, visual acuity, CST, and proportions of SRF and IRF at baseline were similar in both groups. Over 90% patients belonged to white ethnicity and more participants were females.

**Table 1:** Baseline demographic and clinical characteristics across both groups.

	4 loading aflibercept injections *N* = 355	3 loading aflibercept injections *N* = 640
Age (years)		
Mean (SD)	79.1 (7.5)	79.9(8.0)
Male, *n* (%)	154 (43.4%)	238 (37.2%)
Ethnicity		
White	334 (94.1%)	623 (97.3%)
Other	21 (5.9%)	17 (2.7%)
Visual Acuity (ETDRS letters)		
Mean (SD)	57.9 (14.9)	58.1(12.8)
CST (in microns)		
Mean (SD)	450.1 (159.6)	457.3(166.3)
Eyes with IRF fluid in the central 1 mm subfield, *n* (%)	155 (43.7%)	250 (39%)
Eyes with SRF fluid in the central 1 mm subfield, *n* (%)	237 (66.7%)	389 (60.8%)
Eyes with macular IRF, *n* (%)	172 (48.5%)	300 (46.9%)
Eyes with macular SRF, *n* (%)	300 (84.5%)	524 (81.9%)

*CST* central subfield thickness, *ETDRS* Early Treatment Diabetic Retinopathy Study, *IRF* intraretinal fluid, *SD* standard deviation, *SRF* subretinal fluid.

### Fluid resolution outcomes

The proportion of eyes with whole dry macula at 12 weeks was studied in the cohort that received 4 loading injections (*N* = 355). There were 85.1% eyes with no IRF and 77% with no SRF and 67% had neither IRF nor SRF. Table 2 shows the proportion of dry fovea at 4 weeks post 3 loading injections compared to clinical trial data.

**Table 2:** Proportion with fluid resolution at 12 weeks compared to clinical trial data. (4 weeks after 3 loading doses).

	Our study cohort seen at 4 weeks post 3 aflibercept loading doses (*n* = 355)	Pooled TENAYA and LUCERNE cohort -aflibercept arms seen 4 weeks post 3 loading doses (*n* = 664)	Pooled TENAYA and LUCERNE cohort -faricimab arms seen 4 weeks post 3 loading doses (*n* = 665)
No IRF in central 1 mm subfield	299 (84.2%)	85%	88%
No SRF in central 1 mm subfield	286 (80.6%)	79%	88%
No IRF or SRF in central 1 mm subfield	257 (72.4%)	67%	77%

*IRF* intraretinal fluid, *SRF* Subretinal fluid.

**Fig. 1:**
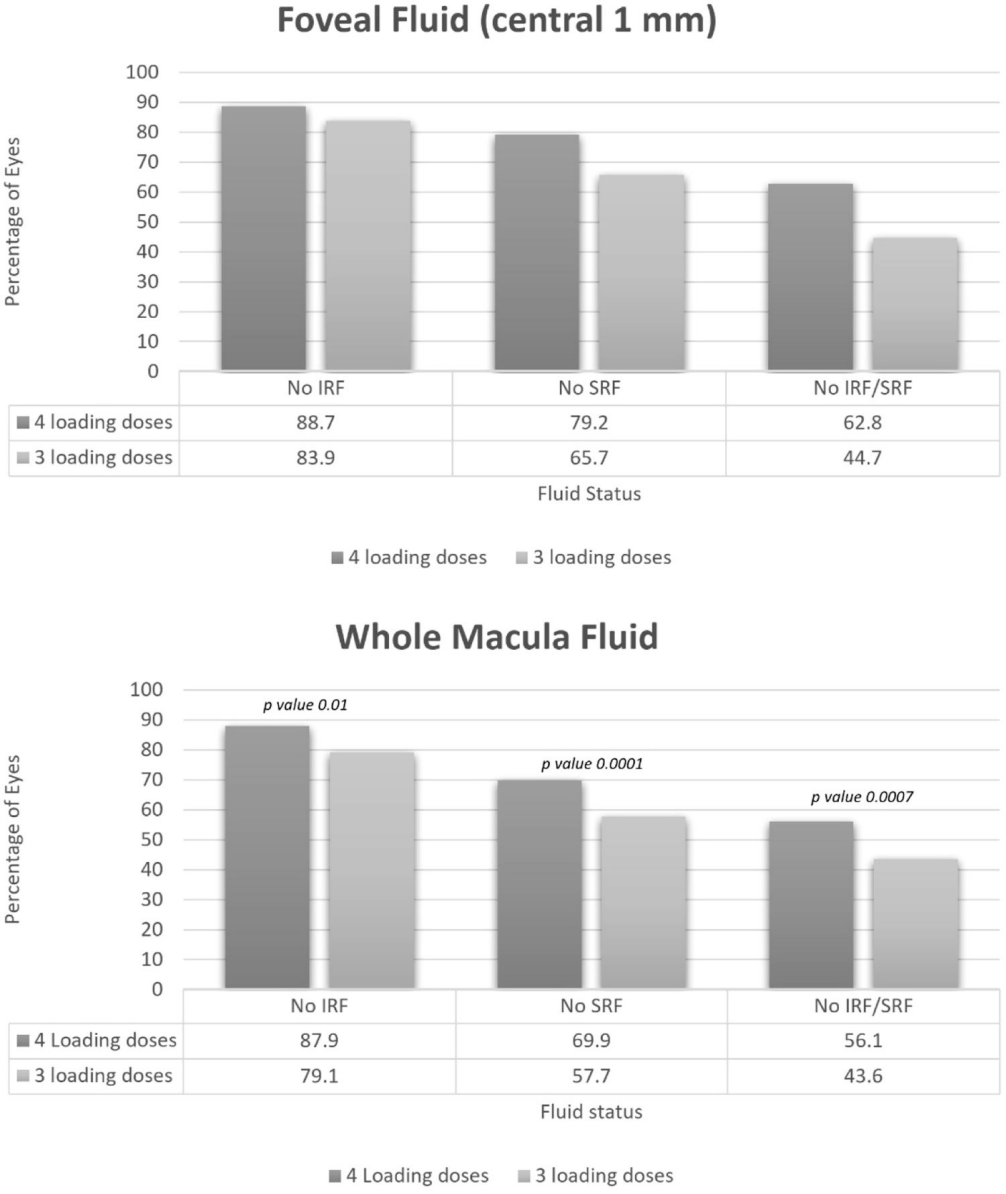
shows outcomes 8 weeks after 4 versus 3 aflibercept loading doses. IRF intraretinal fluid, SRF Subretinal fluid.

Figure 1 shows the outcomes 8 weeks after 4 versus 3 aflibercept loading doses in the central 1 mm and entire macula. The proportion of eyes without IRF (*p* value 0.04), SRF (*p* value 0.0001) or any IRF/SRF (*p* value 0.0001) were significantly higher in the eyes that received 4 loading dose injections.

**Fig. 2:**
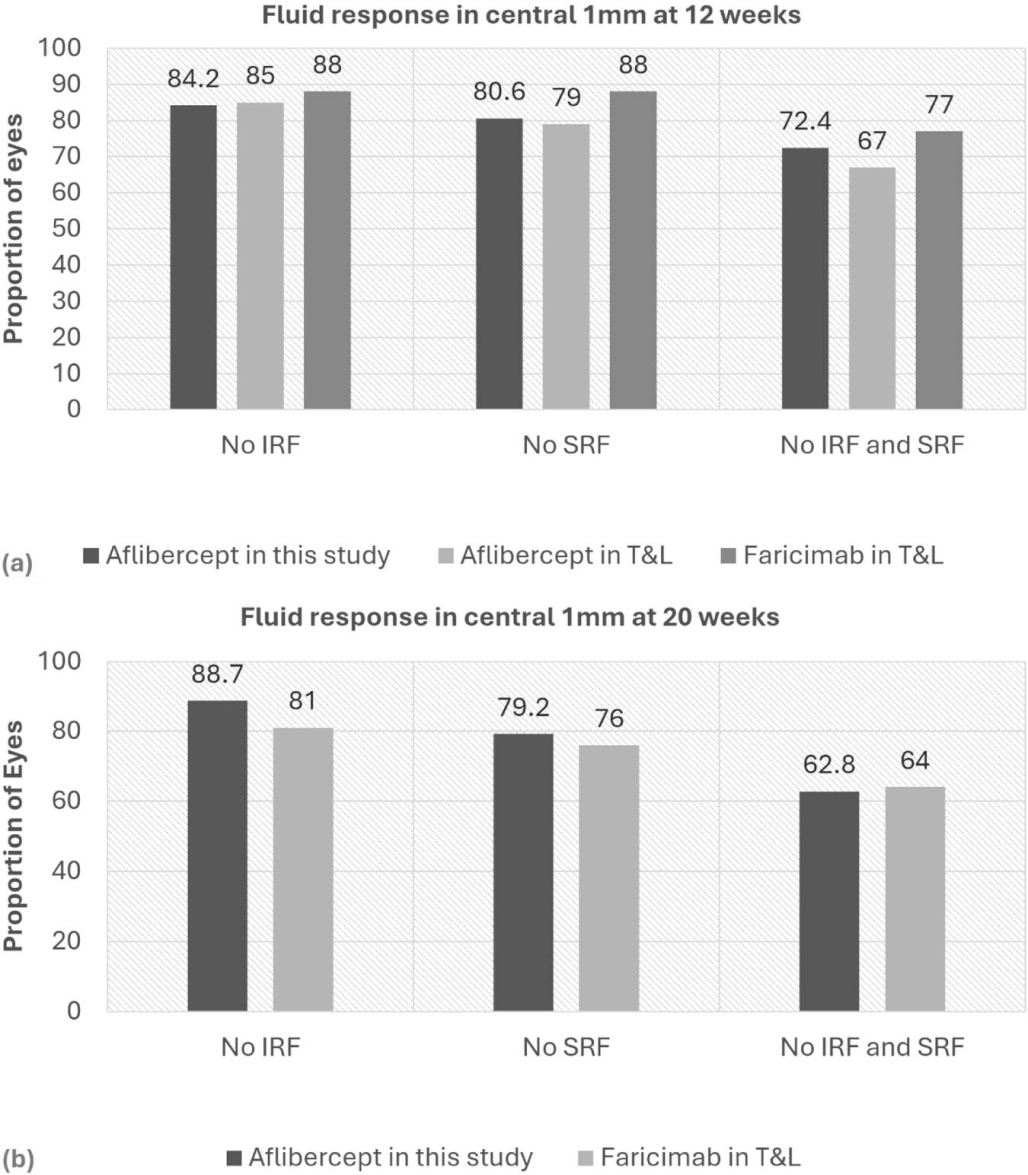
Comparison of fluid response to 3 and 4 loading doses of aflibercept compared to faricimab. Figure **a** shows the fluid response in central 1 mm, 4 weeks after 3 loading doses i.e. at 12 weeks. Figure **b** shows fluid response in central 1 mm, 8 weeks after 4 loading doses i.e. at 20 weeks. IRF intraretinal fluid, SRF Subretinal fluid, T&L TENAYA and LUCERNE.

Figure 2 shows the change in proportion of patients without central 1 mm IRF, SRF or neither IRF nor SRF achieved at week 12 after loading doses of either aflibercept or faricimab. The proportions declined across all groups irrespective of the drug used, except for the resolution of IRF 8 weeks after 4 loading doses of aflibercept in our study. IRF resolution 8 weeks after 4 loading doses was 4.5% higher than 4 weeks after 3 loading doses of aflibercept.

### Visual outcomes

Table 3 shows the comparison of mean change in VA at 8 weeks following 3 loading doses versus 4 loading doses of aflibercept across resolution patterns across various fluid compartments. There was no statistically significant difference in change in mean VA in all the groups.

**Table 3:** Comparison of mean change in VA in at 8 weeks following 3 loading doses versus 4 loading doses of aflibercept.

	3 loading doses of aflibercept *N* (%) Mean VA (SD)	4 loading doses of aflibercept *N* (%) Mean VA (SD)	*P* value
Whole cohort	640 (100%)	355 (100%)	0.09
	+4.0 (11.3)	+5.4 (13.3)	
No IRF at central 1 mm	537 (83.9%)	315 (88.7%)	0.14
	+4.2 (11.3)	+6.1 (13.0)	
No SRF at central 1 mm	421 (65.7%)	281 (78.6%)	0.08
	+4.6 (10.9)	+5.9 (13.3)	
No IRF and SRF at central 1 mm	286 (44.7%)	223 (62.8%)	0.32
	+5.0 (11.0)	+5.8 (13.0)	

*IRF* intraretinal fluid, *SD* Standard deviation, *SRF* Subretinal fluid *VA* Visual acuity.

## Discussion

This study was conducted to determine the patterns of fluid resolution in the central subfield as well as whole macula 6x6mm scans after loading doses of aflibercept injections in exudative AMD in the real world to answer three main questions. Firstly, what is the drying effect of aflibercept 4 weeks after 3 loading injections in clinical practice compared to randomised controlled trials? Secondly, what is the effect of a fourth loading dose of aflibercept on fluid resolution compared to three loading doses of aflibercept or four loading doses of faricimab? And finally, the impact of fourth loading dose of aflibercept on visual outcomes?

To answer the first question, our study showed that a dry scan both in the central subfield and in the entire macular scan at 12 weeks (4 weeks after 3 loading aflibercept injections) was similar to that achieved in the aflibercept arms of the VIEW studies and pooled TENAYA and LUCERNE studies respectively, highlighting the generalisability of our study findings [3, 9]. The pattern of fluid resolution in the whole macular scan and central subfield at 12 weeks reveal that SRF is indeed more resistant to resolution to 3 loading doses of aflibercept. The drying effect in the central subfield at 12 weeks in our cohort (72.4%) closely matched aflibercept (67%) and faricimab (77%) at 12 weeks in the TENAYA and LUCERNE trials. In contrast, just over half of the eyes (55.5%) treated with ranibizumab in the VIEW studies had no IRF or SRF, and we hypothesize this effect would be expected with ranibizumab or ranibizumab biosimilars. Therefore, our results suggest that eyes initiated on ranibizumab or ranibizumab biosimilars might need to be re-assessed 4 weeks after 3 loading doses so that eyes with persistent IRF or SRF can be switched to either aflibercept or faricimab [14].

With the understanding that the drying effect of aflibercept after 3 loading doses is comparable to drying effect of 3 faricimab loading injections when assessed at 4 weeks post loading, we studied the outcome 8 weeks post 3 aflibercept injection in our study cohort versus outcome of aflibercept at the same timepoint in TENAYA and LUCERNE. Again, the proportion of eyes that had no fluid (no IRF/SRF) in the central subfield 8 weeks after 3 loading aflibercept injections (i.e., at 16 weeks) in our real-world study cohort was 44.7% and similar to 46% achieved in the aflibercept arm in TENAYA and LUCERNE clinical trial data [9]. These results exemplify that a considerable proportion of eyes may require 4-weekly aflibercept injections after three loading doses.

We then aimed to answer our second question on the effect of fourth aflibercept injection in our study cohort compared to the fourth faricimab injection 8 weeks after loading doses in the pooled faricimab arms of the TENAYA and LUCERNE cohort [9]. We found that the fourth aflibercept injection had a profound drying effect on both IRF and SRF. At 8 weeks post 4 loading aflibercept injections, 62.8% eyes had neither IRF nor SRF in the central subfield, which was significantly higher than the 44.7% achieved at 8 weeks following 3 loading doses. Our indirect comparison also showed that the drying effect of aflibercept was equivalent to that achieved with 4 loading doses of faricimab (64%) in the pooled TENAYA and LUCERNE cohort at the same time-point (20 weeks post-loading). It was also interesting to note that the drying effect on IRF was better for 4 aflibercept injections (88.7%) versus 4 faricimab injections (81%) but the response of SRF was almost similar with aflibercept and faricimab (79.2% versus 76%). This differential response of aflibercept on fluid compartments was observed both at 4 weeks post 3 loading injections and 8 weeks after 4 injections. A similar response was also seen in the aflibercept arms of both VIEW and TENAYA and LUCERNE cohorts [3, 9]. Notably, a similar difference in response of IRF and SRF was observed with faricimab at 8 weeks after 4 faricimab injections.

These findings highlight that SRF is less responsive to both these agents once a longer injection interval than 4 weeks is considered. It could be argued that SRF is more tolerable to treating clinicians as it is not as detrimental to visual outcome as IRF [12]. However, persistent SRF has an adverse effect on visual function. In addition, fluctuation of macular fluid is also a poor visual prognostic indicator [15]. In the current era of testing durability of agents that can suppress exudation in nAMD our study results indicate caution in extending injection intervals in eyes with persistent fluid. A realistic aim of treatment with anti-VEGF therapy is to maintain the anatomic stability that was achieved by the end of the loading phase, rather than extending all patients based on a protocol followed in clinical practice [16, 17].

However, a question that often arises is whether persistent fluid after loading doses affect VA. We examined if the 4^th^ loading dose injection of aflibercept has a bearing on visual acuity. Comparison of mean change in visual acuity 8–10 weeks after three versus four aflibercept loading doses showed no statistically significant difference. Therefore, better anatomical outcomes in terms of fluid resolution in the subfoveal location did not affect the visual outcome in the short term. This disconnect between fluid resolution and VA outcomes suggest that there may be other OCT parameters that contribute to VA change such as subfoveal loss of ellipsoid layer, atrophy, fibrosis and type of macular neovascularisation. Signs of disease activity are also not limited to presence of IRF or SRF alone and have broadened to include subretinal hyperreflectivity and/or presence of pigment epithelial detachment. These imaging biomarkers therefore need to be explored and graded while individualising treatment regimens.

Our study has many strengths. It is the first study showing effect of 4^th^ loading dose of aflibercept in the real world allowing potential comparison with newer agents. The data was collected from four centres across the UK thereby accounting for variations in clinical practice [18]. In addition, we assessed any IRF or SRF across the 6x6mm macular scans as well, instead of limiting to foveal presence of IRF/SRF to reflect UK clinical practice. The limitations for our study include the retrospective design. We also compared real-world data to clinical trial data from VIEW and TENAYA and LUCERNE that recruit patients based on strict eligibility criteria. We also did not analyse visual outcomes in the longer term. However, there are sufficient evidence from clinical trials that visual outcomes of aflibercept is non-inferior to any of the available anti-VEGF agents including faricimab.

## Conclusion

In summary, 4 loading dose injections of aflibercept 2 mg results in a higher proportion of eyes with dry macula when compared to those receiving 3 loading dose injections when assessed at 8 weeks post last injection. The visual outcomes were better in the cohort receiving 4 loading injections; however, this difference was not statistically significant.

### What was known before

- Fluid resolution outcomes post 3 loading doses of aflibercept.

### What this study adds

1. First study to present effect of 4 loading dose injections of 2 mg aflibercept.
2. Four loading injections have a higher proportion of eyes with dry macula at 12 and 20 weeks – both in the entire macula and in the central 1 mm.
3. This effect was significantly better than 3 loading doses of 2 mg aflibercept and comparable to 4 loading doses of faricimab.
4. However, this improved macular drying effect did not translate into significantly improved visual outcomes in these eyes.

## Supplementary information

Figure S1

## Data Availability

The data that support the findings of this study are not openly available due to reasons of sensitivity and are available from the senior author (Sobha Sivaprasad) upon reasonable request.
