# Supplementary material for: The effect of four loading intravitreal aflibercept injections on macular fluid in treatment-naïve neovascular age-related macular degeneration
Source: Eye (Lond). 2024 Jul 6;38(15):3005–10. doi: 10.1038/s41433-024-03214-x (PMC11461640; doi:10.1038/s41433-024-03214-x)
Supplement: Supplementary file 1 — Figure S1 [file 41433_2024_3214_MOESM1_ESM.docx]

Numbers included for the study across 4 centers in the United Kingdom

Inclusion Criteria:

- Treatment naïve wet AMD eye in study eye
- Eyes should have received only aflibercept injections.

**(N = 1104 eyes of 1104 patients)**

# Enrollment

Excluded **(n = 109 eyes of 109 patients)**

Exclusion Criteria:

1. Mandatory visit (baseline or visit 8 weeks post loading dose) scans missing- 52.
2. Wrong diagnosis-10
3. Ungradable-47

*(due to either poor scan quality (</=20dB); >500µ foveal atrophy; Subfoveal Haemorrhage>50% of lesion; Central fibrosis>1000µ)*

#

**N = 995 eyes of 995 patients**

# **Final Analysed** cohort at 1 year

**Group 2 – 3 loading injections of aflibercept**

**N = 640 eyes of 640 patients**

**Group 1 – 4 loading injections of aflibercept**

**N = 355 eyes of 355 patients**

Figure S1: Participant Flow

***Abbreviations**: AMD- Age related macular degeneration; dB- decibel; µ-microns
